# ΔNp63α enhances the oncogenic phenotype of osteosarcoma cells by inducing the expression of *GLI2*

**DOI:** 10.1186/1471-2407-14-559

**Published:** 2014-08-01

**Authors:** Ram Mohan Ram Kumar, Michael M Betz, Bernhard Robl, Walter Born, Bruno Fuchs

**Affiliations:** Laboratory for Orthopaedic Research, Department of Orthopaedics, Balgrist University Hospital, University of Zurich, Zurich, Switzerland

**Keywords:** Osteosarcoma, p63, ΔNp63α, GLI2

## Abstract

**Background:**

ΔNp63, a splice variant of p63, is overexpressed and exhibits oncogenic activity in many cancers including pancreatic and breast cancer and promotes cell survival by inhibiting apoptosis. Despite its role in tumorigenesis, mechanistic activity of ΔNp63 mediated oncogenic function in osteosarcoma is poorly understood.

**Methods:**

The expression levels of p63 isoforms in osteosarcoma cell lines were identified using quantitative techniques. Expression profiling using microarray, siRNA mediated loss-of-function, and chromatin immunoprecipitation assays were employed to identify novel ΔNp63α targets in *p63*-null osteosarcoma SaOS-2 cells that were engineered to express ΔNp63α. The phenotype of SaOS-2-ΔNp63α cells was assessed using wound-healing, colony formation, and proliferation assays.

**Results:**

The comparative expression analyses identified ΔNp63α as the predominant p63 isoform expressed by invasive OS cell lines. Phenotypic analyses of SaOS-2-ΔNp63α cells *in vitro* indicate that ΔNp63α imparted tumorigenic attributes upon tumor cells. Further, we show that in osteosarcoma cells ΔNp63α directly regulated the transcription factor *GLI2*, which is a component of the hedgehog signaling pathway, and that functional interactions between ΔNp63α and *GLI2* confer oncogenic properties upon OS cells.

**Conclusions:**

Here, we report that *GLI2* is the novel target gene of ΔNp63α and that ΔNp63α-*GLI2* crosstalk in osteosarcoma cells is a necessary event in osteosarcoma progression. Defining the exact mechanisms involved in this interaction that mediate the pathogenesis of osteosarcoma promises to identify targets for drug therapy.

**Electronic supplementary material:**

The online version of this article (doi:10.1186/1471-2407-14-559) contains supplementary material, which is available to authorized users.

## Background

Osteosarcoma (OS) is a highly malignant bone tumor with an increased prevalence in children and young adults. OS commonly occurs in the metaphysis of long bones adjacent to the growth plates where bone growth occurs during puberty. Osteosarcomas are generated by malignant transformation of mesenchymal cells, which normally differentiate to osteoid- and bone-forming cells [1]. The majority of patients with newly diagnosed OS suffer from localized disease and up to 70% survive with state-of-the-art treatment, which comprises local surgical control of the primary tumor combined with neoadjuvant multidrug chemotherapy [2]. Unfortunately, 15–30% of OS patients present with metastases at diagnosis and their 5 year survival rate is only approximately 20% regardless of therapy [3]. Thus, the pathogenesis of OS requires further research to develop more effective treatment of metastatic disease.

The *p63* gene, a member of *p53* gene family, encodes the isoforms TAp63 and ΔNp63 [4]. TAp63 and ΔNp63 are transcribed from two distinct *p63* promoters- P1 and P2 and they are differentially spliced at their C- termini to generate the variants α, β, γ, Δ and ε [5]. The “long” isoforms are collectively described as TAp63, contain an N-terminal transactivation (TA) domain and suppress tumorigenesis and metastasis. Mice lacking TAp63 develop spontaneous carcinomas, sarcomas, tumors of the bone, fat, and cartilage supporting the conclusion that TAp63 is a tumor suppressor [6]. In contrast, the “short” isoforms collectively described as ΔNp63, which lack the TA domain, exert oncogenic properties and overexpression of ΔNp63 promotes cell proliferation *in vitro* and tumor growth *in vivo* of many cancers [7]. The proteins encoded by *p63,* unlike *p53*, mediate the development and differentiation of epithelial surfaces and act as oncogenes or tumor suppressors depending on the cell type [8].

Studies on epigenetic modifications and expression of genes involved in cell proliferation and metastasis indicate that genomic instability is a hallmark of OS. Studies on the role of ΔNp63 in the regulation of cell proliferation and in the cell cycle led to the hypothesis that up-regulation of ΔNp63 expression might counteract p53 functions thereby explaining the oncogenic effects of ΔNp63 [5, 7]. However, the tumorigenic effects of ΔNp63 may also be mediated by promoting the transactivation of target genes such as those encoding Lsh, which is a chromatin remodeler, involved in tumorigenesis and stem cell proliferation as well as genes encoding interleukin (IL)- 6 and (IL)-8 that are involved in tumor angiogenesis associated with neuroblastomas and osteosarcomas [9, 10]. ΔNp63α is the most abundant p63 isoform expressed in cancer cells [11] and is overexpressed in squamous cell carcinoma which enhances cell growth by counteracting p53-mediated growth arrest and apoptosis [12].

ΔNp63 isoforms are predominantly expressed in bladder carcinomas, non-small cell lung cancers and liver cancers among others, indicating their role in carcinogenesis. These findings are surprising, because their expression is undetectable in breast and prostate adenocarcinomas [13]. Moreover, ΔNp63α is implicated in OS progression and is involved in tumor angiogenesis [10]. Independent of p53 mutational status, frequent up-regulation of ΔNp63α in OS increases the levels of IL-6 and IL-8 that induce increased phosphorylation of signal transducer and activator of transcription 3 (STAT-3) to promote oncogenesis. Moreover, the levels of ΔNp63α in lung metastases of patients with OS are higher compared with corresponding primary lesions in the same patients [10].

*In vivo* studies using short hairpin RNA mediated knockdown of ΔNp63 expression showed that the tumor volume in mice decreased significantly compared with control mice carrying tumors transduced with control shRNA [10]. However, the mechanism that regulates the expression of *p63* in OS particularly the ΔNp63 isoforms is unknown. Here, we provide new insights into the mechanism that controls the ability of ΔNp6α to enhance the malignant phenotype of OS cells and show that the expression of *GLI2*, a transcriptional activator and mediator of the Hedgehog (HH) signaling pathway, is regulated by ΔNp63α.

## Methods

### Cell culture and reagents

The human OS cell lines SaOS-2 (HTB-85), U2OS (HTB-96), HOS and 143B were obtained from the American Type Culture Collection (Manassas, VA, USA). The SaOS-2, LM5 was provided by E.S. Kleinerman (M.D. Anderson Cancer Center, Houston, TX, USA). Hu09 cells and the Hu09-M132 subline were provided by Dr. M. Tani (National Cancer Center Hospital, Tokyo, Japan), MG-63 cells were provided by Dr. G. Sarkar (Mayo Clinic, Rochester, MN, USA) and the MG-63 subline M8 was provided by Dr. W.T. Zhu (Tongji Hospital, Huazhong University of Science and Technology, Wuhan, China). All cell lines were cultured in Dulbecco’s Modified Eagle Medium (DMEM containing 4.5 g/l glucose)/Ham F12 (Invitrogen, Carlsbad, CA, USA) (1:1) supplemented with 10% fetal calf serum (FCS). All cells were cultured at 37°C in a humidified atmosphere of 5% CO_2_. BxPC3 cell lines were kindly provided by Dr. Corina Kim Fuchs (Inselspital Bern, Switzerland). The cells were cultured in RPMI 1640 medium containing 10% FCS and then incubated at 37°C in an atmosphere containing 5% CO2. The invasive cell lines LM5 and M132 were derived from the SAOS and HUO9 cell lines by repeated intravenous injections of mice with cells isolated sequentially from lung metastases. The 143B cell line was generated by transforming HOS cells with K-ras and the M8 cell line was generated by *in vitro* sub cloning of MG63 cells [14–17]. GANT61 was purchased from Bio vision Inc. (San Francisco, USA). For p63 knock-down experiments 143B and M132 cells were transiently transfected with Lipofectamine LTX reagent (Life Technologies, USA).

### Tissue microarray construction

All the tissues were fixed in 4% formaldehyde and embedded in paraffin. Paraffin-embedded donor tissue blocks were sampled using a Manual Tissue Arrayer 1 instrument (Beecher Instruments, Silver Spring, MA, USA). Sections were cut for hematoxylin-eosin staining and histopathologically representative tumor regions were used for preparation of TMA blocks. After the TMA construction, sections were cut from the “donor” blocks comprising of 61 tumor biopsies and 55 tumor resections having sufficient material available. Sections (5 μm) of the tissue array block were cut and placed on polylysine-coated glass slides and processed for immunohistochemical staining (IHC) with rabbit anti-ΔNp63 (1:500). The tissue cores were graded by two independent trained researchers. The cores were considered negative if less than 50% of the cells were stained with ΔNp63 and if the staining is seen in more than 50% of the cells, the cores were considered as positive for ΔNp63.

### Retroviral transduction of cell lines

Constructs for stable constitutive expression of TAp63α, TAp63γ, ∆Np63α and ∆Np63γ were provided by Maranke Koster (University of Colorado, Denver, USA) and were cloned using the pQCXIH vector. Retroviral particles containing the described constructs were produced in HEK293-T cells according to a published method [18]. Briefly, HEK293-T cells were cultured in Advanced D-MEM medium (GIBCO) supplemented with 2% fetal calf serum and a culture additive containing 0.01 mM cholesterol (Sigma-Aldrich), 0.01 mM egg yolk lecithin (Serva Electrophoresis GmbH, Heidelberg, Germany) and 1x chemically defined lipid concentrate (GIBCO) (transfection medium). The cells were co-transfected using the calcium phosphate method with the following three plasmids: a retroviral expression vector together with the two helper plasmids pVSV-G (Clontech), encoding the G-glycoprotein of the vesicular stomatitis virus, and pHit60 encoding the retroviral gag and pol genes (provided by Dr. Christian Buchholz, Paul-Ehrlich- Institute, Langen, Germany). Fourteen hours after transfection the medium was replaced with fresh transfection medium. The supernatant containing each recombinant retrovirus was collected 48 h after transfection, filtered through a 0.45 μm syringe filter and stored in aliquots at - 80°C.

### cDNA synthesis and expression analysis

Total RNA was isolated from cell lines using an RNeasy mini kit (Qiagen, Valencia, CA, USA), and 1 μg of RNA was used as template for cDNA synthesis using a High-Capacity cDNA reverse transcription kit (Applied Biosystems, Foster City, CA, USA). Semi-quantitative PCR amplification of each p63 isoform was performed using primers described previously [19]. PCR was performed as follows: denaturation at 94°C for 3 min, 35 cycles of incubation at 94°C for 40 s and at 55°C for 40 s and incubation at 72°C for 4 min. For real time PCR (qRT-PCR) analysis three independent RNA preparations from each cell lines were reverse transcribed in a final volume of 10 μl. qRT-PCR was performed using the StepOne Plus Real- Time PCR system (Applied Biosystems, USA) in 96 well plates. Primers (Additional file 1) used to amplify cDNAs were designed using NCBI-primer software (http://www.ncbi.nlm.nih.gov/tools/primer-blast/). PCR amplification of individual qRT-PCR reactions were carried out in triplicate. The cDNA and appropriate primers were added to Power SYBR Green PCR master mix (Applied Biosystems, USA) and the samples were pre-incubated as follows: 50°C for 2 min and at 95°C for 10 min, 40 cycles at 95°C for 15 s and at 60°C for 1 min. The threshold of Ct values was set to 0.325. To verify the amplification of a single product in any of the PCR reactions, a melting curve was generated and analysed after every run. Relative expression levels were calculated by the comparative cycle threshold (ΔΔCT) method and were normalized to *GAPDH* expression.

### Microarray analysis

Complementary RNA preparation and array hybridization were performed at the Functional Genomics Center of the University of Zurich, using the Agilent SurePrint Human Gene Expression 8 × 60K array. Genes with a False Discovery Rate with an adjusted *P* value < 0.005 and a fold change (fc) > 2 were considered differentially expressed. To identify specific genes expressed in samples transfected with ΔNp63α compared with the empty vector, we used a computer algorithm that allowed us to select genes exhibiting ≥ 3 fold changes. Genes expressed at ≥ 3 fold levels in SaOS-2-EV and SaOS-2- ΔNp63α cells were aligned using a computer algorithm and Microsoft Excel. Three replicas of each sample were analysed and genes that showed a common pattern of fc in each of the three individual experiments were selected for further analysis. Enrichment analysis was performed on the web platform, Database for Annotation, Visualization and Integrated Discovery (DAVID 2.0; http://david.abcc.ncifcrf.gov/). Functional annotates acquired from the list of regulated genes and the probe sets that identified multiple transcripts were removed. From DAVID analysis, we selected the pathways of Kyoto Encyclopaedia of Genes and Genomes (KEGG pathway) that include a compilation of the network of molecular interactions in cells. The micro array data have been deposited in the Gene Expression Omnibus (Accession No: GSE54942).

### Western blot analysis

Cells were lysed by agitation on a rotating platform at 4°C for 1 hour in lysis buffer containing 50 mM Tris/ HCl (pH 7.5), 150 mM NaCl, 1% NP40, 0.5% deoxycholic acid, 0.1% sodium dodecyl sulfate (SDS), 1 mM dithiothreitol, 1 mM phenylmethylsulfonyl fluoride , and 10 mg/mL aprotinin. Cellular debris was removed by centrifugation at 16,060 × *g* and 4°C for 20 min. An equal amount of protein of each cell extracts was subjected to 10% SDS-PAGE and the proteins were transferred using semi-dry blotting to Hybond-ECL membranes (GE Healthcare, Glattbrugg, Switzerland). Western blot analysis was performed by probing the membrane with the following antibodies at the indicated dilution as follows: anti-rabbit ΔNp63 (1:500; provided by Dr. James DiRenzo, Dartmouth Medical School), anti- goat *GLI2* (1:200, Santa Cruz Biotechnology, CA, USA), anti-rabbit *GAPDH* (1:3000; Santa Cruz Biotechnology) and species specific horseradish peroxidase conjugated secondary antibodies (Santa Cruz Biotechnology). Peroxidase activity was detected using the Immobilon chemiluminescence substrate (Millipore, Billerica, MA, USA) and the signals were recorded using a VersaDoc Imaging System (Bio-Rad, Hercules, CA, USA).

### RNA interference

*p63* siRNA and a non-targeting control siRNA were purchased from Santa Cruz (Santa Cruz Biotechnology). 143B and M132 (1 × 10^5^ cells) were seeded in a 6-well plate cultured to 70% confluence and then transfected for 48 h with 20nM p63 siRNA or control siRNA with Lipofectamine LTX reagent purchased from Invitrogen (Life Technologies, USA). Transfections were carried out according to the manufacturer’s instruction. The efficiency of knock-down was analysed by western blotting.

### Wound healing assay

SaOS-2-TAp63, SaOS-2- ΔNp6α and SaOS-2-EV cells were grown to confluence, and cell motility was determined in an *in vitro* wound healing assay as described previously [20]. Motility was determined from the difference between the wound width at 0 and 24 h and was calculated from measurements of defined areas of images of the wounds taken with an AxioCam MRm camera connected to a Zeiss Observer.Z1 inverted microscope at 4× magnification. The motility of SaOS-2-EV cells or of untreated cells was defined as 100%.

### Cell proliferation assay

Wells of 96 well plates were seeded with 3 × 10^3^ cells that were allowed to adhere and grow overnight. The cells were then incubated with 10 μl per well of WST-1 reagent (Roche, Basel, Switzerland) for 3 h and cell metabolic activity per well was determined according to a published procedure [21].

### Soft-agar colony formation assay

The experiments were carried out in six-well cell culture plates containing 1.5 ml per well of 0.5% base agar in cell culture medium supplemented with penicillin, streptomycin and ampicillin (PSA). Single cell suspensions (2 × 10^4^cells per well) were prepared and added (1.5 ml per well of cell culture medium containing 0.35% agar and PSA ) added on top of the base agar and incubated in a humidified atmosphere containing 95% air and 5% CO_2_ at 37°C. Twenty-four hours later the top agar was overlaid with 2 ml/well cell culture medium containing PSA. Cells were incubated for 16 days and the medium was changed at 3-day intervals. Colonies were stained with 0.005% crystal violet. Images were acquired using a Nikon Eclipse E600 camera.

### Immunofluorescence

Cells grown on glass coverslips were fixed with 3.7% formaldehyde in phosphate-buffered saline (PBS) for 15 min and permeabilized with 0.2% Triton X-100. The fixed cells were treated with blocking solution (2% fetal bovine serum in PBS) and stained with rabbit anti-ΔNp63 (1:500) together with goat anti-*GLI2* (1:400) in blocking solution incubated at 4°C overnight. The next day, the cells were washed 3 times with PBS, and stained with Dylight 594-labeled donkey anti-rabbit, or Cy5-labeled donkey anti-goat secondary antibodies (Jackson Immuno Research Laboratories, Inc., Baltimore, Pike West Grove, USA) at room temperature for 1 h. Nuclear DNA was stained with 0.2 μg/ml 4′,6′-diaminidino-2-phenylindole (Molecular Probes Inc., Eugene, USA). Fluorescence imaging was performed using a confocal laser scanning microscope (SP5, Leica, Heerbrugg, Switzerland) equipped with a Plan-Apochromat 63 × NA 1.4 oil immersion objective.

### Cell cycle analysis

Cell cycle analysis was performed at the Flow Cytometry Facility at ETH Zurich. The cells were seeded in 10 cm dishes at a density of 1.0 × 10^6^ cells per dish. Forty-eight hours after GANT61 treatment SaOS-2-ΔNp6α, SaOS-2-EV and 143B cells were treated with trypsin, collected by centrifugation and washed with PBS. Cells were then fixed in ice cold 70% (v/v) ethanol at 4°C, washed with PBS and then resuspended in 500 μl of ice cold PI/RNase Staining Buffer (BD Pharmingen AG, Allschwil, Switzerland) followed by incubation at 37°C for 30 min in the dark. The samples were analysed using a fluorescence activated cell sorter (FACS) (Calibur, BD, USA). Doublets were excluded and the percentage of cells present in each phase of the cell cycle was calculated using FlowJo software (Ashland, USA).

### Chromatin immunoprecipitation (ChIP) assay

To identify whether ΔNp6α binds to the *GLI2* promoter, chromatin immunoprecipitation (ChIP) was performed using the Millipore ChIP Assay kit (Temecula, CA, USA) according to the manufacturer’s protocol. The cells (1 × 10^7^) were cross-linked with formaldehyde and fragmented by sonication to yield chromatin fragments of ~500 bp determined using agarose gel electrophoresis. The cell extracts were treated with Protein A/G agarose beads before incubation overnight with 10 μg of the ΔNp6α antibody or the corresponding IgG. The antibody-DNA complexes were incubated with Protein A/G agarose beads and cross links were reversed at 65°C in a rotating incubator for 8 h. Immunoprecipitated DNA was analysed by using qRT-PCR with GLI2 specific primers as follows: GLI2-1; forward; 5′- GCCACCTGCGTGCTAGA-3′ and reverse; 5′-GGCCAATGCAACTTTACC-3′, GLI2-2; forward; 5′- ACTCCCATCAATGAGACTTCG-3′ and reverse; 5′- AAGAGAGGGGACCGAGAGG-3′. PCR conditions were as follows: initial denaturation at 94°C for 5 min, 40 cycles at 95°C for 30 s, 62°C for 30 s, 72°C for 50 s and final extension at 72°C for 10 min.

### Statistical analysis

Data from triplicate samples were analysed using GraphPad Prism5 software (GraphPad Software, Inc.; La Jolla; CA, USA) and the differences between means were evaluated using the Student’s t- test and *P* <0.05 was considered to indicate a significant difference. Kaplan Meier survival analysis was performed using PASW Statistics 18 (IBM Corporation; New York; USA). The results are presented as the mean ± standard error of the mean (SEM).

### Ethics statement

All of the studies involving human participates were fully encoded to protect patient confidentiality and were utilized under a protocol approved by the local ethic committee (approval reference number StV 41–2005).

## Results

### ΔNp63α is the predominant *p63*isoform expressed by invasive OS cells

**Figure 1:**
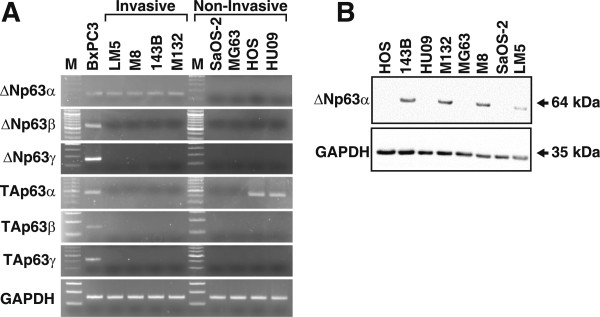
**Analysis of the expression of p63 isoforms in human OS cell lines.** The human parental low metastatic (non-invasive) SaOS-2, MG63, HOS and HUO9 cell lines and the respective metastatic (invasive) LM5, M8, 134B and M132 sublines were analysed. **A**. mRNA levels of p63 splice variants in the indicated OS cancer cell lines were determined using semi quantitative PCR. BxPC3 cells served as the positive control and *GAPDH* mRNA levels were used as loading controls. M, molecular size marker lane. **B**. Protein levels of ΔNp63α in the indicated OS cell lines were detected using an antibody specific for ΔNp63.

Human *p63* generates the- α, β, and γ isoforms of TAp63 and ΔNp63 with distinct biological functions. To identify the *p63* isoforms expressed by human OS cell lines, we conducted an expression analysis of various OS cell lines that represent heterogeneity of tumors *in situ*. Transcripts encoding TAp63α were only detected in the non-invasive human cell lines (HOS and HU09) and were undetectable in the invasive OS cell lines (LM5, M8, 143B, M132) (Figure 1A). In contrast, transcripts encoding ΔNp63α were expressed exclusively in the invasive OS cell lines. Transcripts encoding the β and γ isoforms of TAp63 and ΔNp63 were undetectable in the low and high metastatic cell lines.

The specificity of the ΔNp63 isoform-specific antibody was confirmed by western blotting analysis of lysates prepared from SaOS-2 cells stably transfected with vectors expressing TAp63α, TAp63γ, ΔNp63α, ΔNp63γ or with the empty vector. SaOS-2 cells did not detectably express endogenous p63 or p53 and their isoforms. Transfected cells expressed ΔNp63α (64 kDa) and ΔNp63γ (44 kDa) and the ΔNp63 antibodies did not cross-react with TAp63 isoforms (Additional file 2). Using this ΔNp63 specific antibody we analysed extracts from our panel of invasive (LM5, M8, 143B and M132) and the respective parental non-invasive (SaOS-2, MG63, HOS and HU09) OS cell lines and determined that ΔNp63α was expressed only in the invasive OS cell lines (Figure 1B). We compared the levels of ΔNp63α and TAp63α in SaOS-2- ΔNp63α and SaOS-2-TAp63 cells as well as the SaOS-2 cells transfected with the empty vector and with cells that endogenously express the respective isoform of p63 (143B cells, ∆Np63α; HOS cells, TAp63). The ectopic and endogenous proteins were expressed at relatively high levels in the respective cell lines. Specifically, there was a 90% increase in SaOS-2- ΔNp63α and SaOS-2-TAp63α protein levels in transduced and control cells compared with SaOS-EV cells (Additional file 3).

### ΔNp63α overexpression in resections of osteosarcoma patients correlates with worse prognosis

Given the implication of ΔNp63α in tumorigenesis, immunohistochemical analysis of ΔNp63α was carried out to determine whether any correlation of ΔNp63α expression is related with clinical outcome in OS patient samples. We analysed the OS tissue microarray (TMA) based on a two-level grading scheme as described in the materials and methods. ΔNP63 was expressed in 77% of all the biopsies and in 53% of the resections. Using Kaplan–Meier analysis we found no significant correlation between ∆Np63 expression in biopsies and survival of patients (log-rank test, *P* = 0.727) (data not shown). However, the prognosis was worse for patients with high levels of ∆Np63 in resections compared with patients with low or undetectable expression in resected tumor tissue (Additional file 4; log-rank test, *P* = 0.000005).

### ΔNp63α mediates the oncogenic phenotype of OS cells *in vitro*

**Figure 2:**
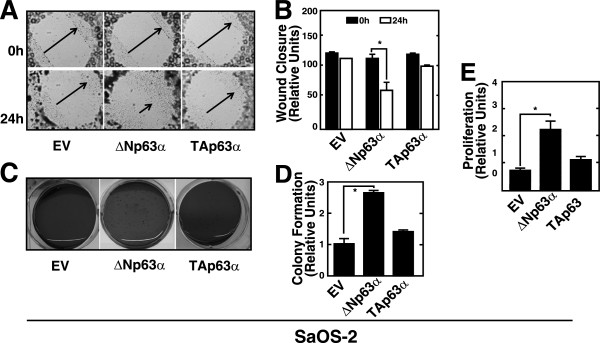
**Overexpression of ΔNp63α enhanced the malignant phenotype of SaOS-2 cells. A**. Analysis of the motilities of SaOS-2- ΔNp63α, SaOS-2-EV and SaOS-2-TAp63α cells in a wound healing assay. Representative photomicrographs of scratch-wounds at the indicated time points after wounding. Arrows indicate the wound width. **B**. Quantitative analysis of wound closure in cultures of SaOS-2-EV, SaOS-2-ΔNp63α and SaOS-2-TAp63α cells. **C**. Analysis of anchorage-independent growth in soft agar of SaOS-2-ΔNp63α, SaOS-2-TAp63α and SaOS-2-EV cells. Representative images of colonies stained with 0.005% crystal violet. **D**. Quantitative analysis of colony formation in soft agar. The numbers of colonies formed by SaOS-2-EV cells were defined as 1. **E**. Analysis of proliferation of SaOS-2-ΔNp63α cells, SaOS-2-EV and SaOS-2-TAp63α cells using a WST assay. Data shown in 2 B, 2 D and 2E represent the mean ± SEM of three independent experiments ( *P < 0.05).

To investigate the effects of ΔNp63α expression in OS, we engineered the non-invasive human osteoblastic *p63*^*-/-*/^/*p53*^*-/-*^ null SaOS-2 OS cell line to stably express ΔNp63α. Because SaOS-2 cells do not express p63 or p53, we asked whether ectopic expression of ΔNp63α in SaOS-2 cells (SaOS-2-ΔNp63α) would reveal its functional significance in OS. SaOS-2 cells transfected with empty vector (SaOS-2-EV) or the TAp63α expression construct (SaOS-2-TAp63α) served as controls. We tested whether ΔNp63α expression altered cell motility, proliferation, or both of non- invasive osteoblast cell lines. The result of the *in vitro* wound-healing assay demonstrated that ΔNp63α enhanced cell motility towards the wound area compared with SaOS-2-TAp63α or SaOS-2-EV cells. The motility of SaOS-2-ΔNp63α cells was significantly higher (*P <* 0.05) than that of SaOS-2-TAp63α and SaOS-2-EV cells (Figure 2A and B). Because anchorage-independent growth characterizes oncogenic transformation and metastatic potential is indicated by the ability of cells to form small colonies in soft agar, we asked whether ΔNp63α regulated anchorage- independent growth. The number of colonies formed by SaOS-2-ΔNp63α cells was higher by a factor of 2.6 (*P* < 0.05*)* compared with SaOS-2-TAp63α or SaOS-2-EV cells. This demonstrated that ectopic expression of ΔNp63α in SaOS-2 cells facilitated anchorage-independent growth (Figure 2C and D).

We next compared the rates of proliferation of SaOS-2-ΔNp63α cells with that of SaOS-2-TAp63α and SaOS-2-EV cells and found that SaOS-2-ΔNp63α cells proliferated more rapidly (*P <* 0.05) (Figure 2E). Thus, ectopic expression of ΔNp63α in SaOS-2 cells significantly enhanced the expression of phenotypic markers of malignancy.

### ΔNp63α-mediated transcript ensemble in osteosarcoma cells

**Figure 3:**
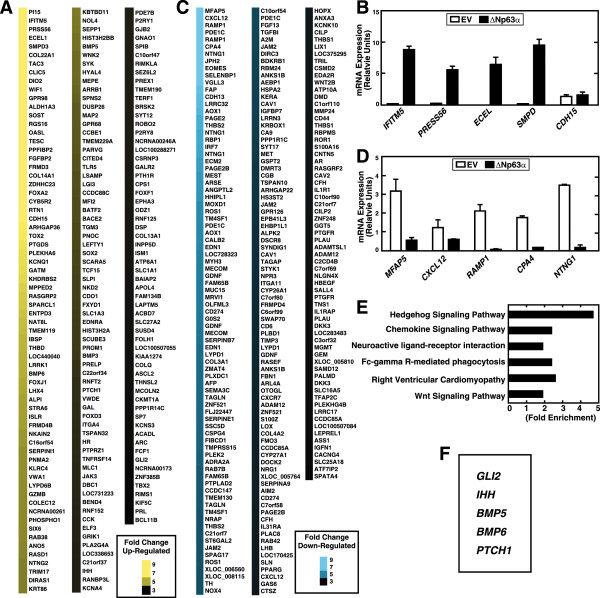
**Microarray analysis to detect differential gene expression in SaOS-2- ΔNp63α cells.** Hierarchical clustering of differentially expressed genes revealed by microarray analysis in SaOS-2- ΔNp63αcells compared with SaOS-2-EV cells. The heat maps depict the fold-change of up- **(A)** and down-regulated **(C)** genes in SaOS-2-ΔNp62α compared with SaOS-2-EV cells in descending order of 10 to 3. Quantitative RT-PCR validation of the microarray results obtained for indicated up **(B)** and down-regulated **(D)** genes. **E**. Transcript enrichment in SaOS-2-ΔNp63α cells grouped by functional related terms from the KEGG pathway analysis. **F**. Genes enriched in Hh signaling pathway are highlighted.

Because ΔNp63α was specifically expressed in invasive OS cell lines and contributed to their oncogenic phenotypes, we analysed the ΔNp63α regulated transcriptome in OS cells to facilitate the identification of genes and signaling pathways that impart the ΔNp63α-mediated oncogenic phenotype of OS cells. Therefore, we compared the global gene expression pattern of SaOS-2-ΔNp63α and control SaOS-2-EV cells. Both cell lines were grown under similar conditions and total RNAs from exponentially growing cells were subjected to microarray analysis. The microarray analysis revealed major differences in the transcriptional profile of SaOS-2-ΔNp63α cells compared with that of the control cells. Thus, 196 and 230 genes were up- or down- regulated by factors of 3 respectively. A heat map showing the differential expression in descending order of 10 and 3 fold up and down-regulated genes respectively is shown in Figure 3A and C.

To validate the microarray data, we selected 10 genes that were highly differentially expressed (Figure 3B and D) and analysed their expression using qRT-PCR as well as with DAVID to reveal their functions. Analysis using the KEGG database yielded specific signaling pathways, molecular functions, and biological processes that are involved the network of differentially expressed genes (Figure 3E and F). Hedgehog signaling genes were enriched by a factor of 5 and the expression of Hh signaling pathway components *GLI2, BMP4, BMP5, PTCH1 and IHH* were highly regulated by ΔNp63α.

### ΔNp63α induces GLI2 expression in OS cells by binding to the *GLI2*promoter

**Figure 4:**
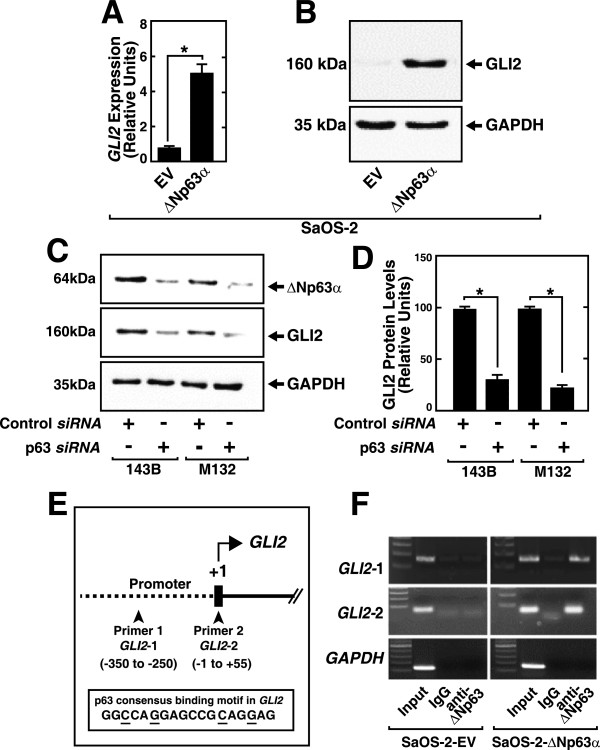
**ΔNp63α activates** ***GLI2*** **transcription. A**. Real time PCR analysis of total RNA isolated from SaOS-2-ΔNp63α and SaOS-2-EV cells shows upregulation of *GLI2* expression in SaOS-2-ΔNp63α cells. *P value < 0.05 (n = 3). **B**. Western blot analysis of cell lysates prepared from SaOS-2-EV and SaOS-2-ΔNp63α cells show the elevated expression of GLI2 in SaOS-2-ΔNp63α cells. **C**. Western blot analysis of ΔNp63α and GLI2 expression in lysates prepared from 143B and M132 cells after transfection with control or p63 siRNAs. **D**. Quantification of relative GLI2 expression is shown in panel D. *P value < 0.05 (n = 3). **E**. The *GLI2* promoter and transcription initiation region and p63 consensus-binding site (essential sequences underlined) probed using chromatin immunoprecipitation (ChIP). **F**. ChIP analysis of occupancy of the *GLI2* promoter by ΔNp63α using control IgG or an antibody against ΔNp63 (anti- ΔNp63) in SaOS-2-EV and SaOS-2-ΔNp63α cells as indicated. Amplification of the *GLI2* and *GAPDH* promoter regions using *GLI2-1* and *GLI2-2* primer sets was analysed using agarose gel electrophoresis. Input control lanes, IgG and *GAPDH* controls are shown.

The results of the transcriptional analysis presented above supports the conclusion that ΔNP63α specifically activates Hh signaling in OS cells. Moreover, *GLI2* is implicated in OS and targeting *GLI2* has been suggested for treating OS. We next investigated whether ΔNp63α regulates the transcription of *GLI2*. We conducted qRT-PCR analysis of SaOS-2-ΔNp63α cells and found that GLI2 mRNA levels were higher by a factor of 4.5 compared with SaOS-2-EV cells (Figure 4A), which is consistent with the microarray data and the western blotting analysis shown in Figure 4B. We next determined the effect of a *p63* siRNA on the OS cell lines (143B and M132) that express high levels of endogenous ΔNp63α. The level of ΔNp63α protein was decreased by approximately 80% in *p63* siRNA-transfected 143B and M132 cells compared with cells transfected with control siRNA (Figure 4C) and was accompanied by a decrease in the level of endogenous GLI2 (Figure 4C and D). In contrast, the level was unchanged in cells transfected with control siRNA. We determined whether TAp63α also regulate GLI2 expression by using siRNA approach to inhibit p63 expression in OS cells (HOS, HU09) that express endogenous TAp63. GLI2 levels did not change when TAp63 expression was inhibited as shown by the western blot (Additional file 5) thereby indicating that GLI2 is not regulated by TAp63.

We asked whether the silencing of ΔNp63α altered the levels of expression of genes that were identified as up-regulated using microarray analysis. We selected the top three genes (*IFITM5*, *SMPD3*, and *ECEl1*) that were up-regulated in SaOS-2-ΔNp63α cells. 143B and M132 cells were selected which expressed endogenous ΔNp63α and p63 expression was inhibited using the cognate siRNA. There was no significant difference in the mRNA levels of IFITM5, SMPD3, and ECEl1 between transfected and control cells (Additional file 6). These data may indicate that those genes are indirectly regulated by ΔNp63α.

We next analysed whether ΔNp63α bound to the G*LI2* promoter. We identified the p63 binding consensus sequence (T/A) A (T) ACA (T) TGT (T/A) T [22] in the promoter region of GLI2 (Figure 4E). To determine whether ΔNp63α bound the GLI2 promoter, we performed ChIP analysis of SaOS-2-ΔNp63α cells using primer sets that amplify the *GLI2* promoter regions harboring the p63 consensus sequence (primer GLI2-1) and the TSS sites (primer GLI2-2) (Additional file 7). The results of ChIP experiments demonstrated that ΔNp63α bound specifically to the *GLI2* promoter region (Figure 4F). The binding specificity was indicated by the controls using the GAPDH promoter and non-specific IgG and the lack of detection of the ΔNp63α-GLI2 promoter complex in SaOS-2-EV cells.

**Figure 5:**
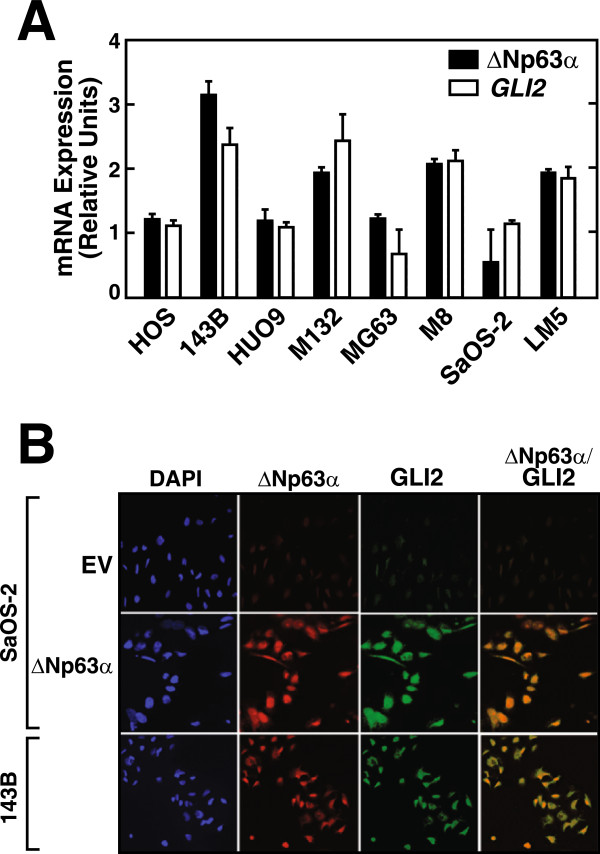
**Analysis of ΔNp63α and GLI2 expression in OS cell lines. A**. qRT PCR analysis of ΔNp63α and *GLI2* mRNA expression in a panel of OS cell lines as indicated. *GAPDH* mRNA was used as an internal control. **B**. Immunocytochemical analysis of ΔNp63α and GLI2 expression in SaOS-2-ΔNp63α, SaOS-2-EV and 143B cells.

The coordinate regulation of the expression of GLI2 and ΔNp63α in invasive and the cognate parental non- invasive OS cell lines was further confirmed by the results of qRT-PCR analysis (Figure 5A). The levels of mRNAs encoding ΔNp63α and *GLI2* were up-regulated only in the invasive cell lines. To gain further insight into the functional correlation between ΔNp63α and *GLI2*, we analysed their expression in 143B cells that expressed endogenous ΔNp63α and GLI2 and in SaOS-2-ΔNp63α cells. Double-immunofluorescence assays detected ΔNp63α and GLI2 in both cell lines suggesting the presence of a ΔNp63α-GLI2 signaling axis in OS cells (Figure 5B). Neither protein was detected in SaOS-2-EV cells.

### *GLI2*mediates the oncogenic phenotype of ΔNp63α in OS cells

**Figure 6:**
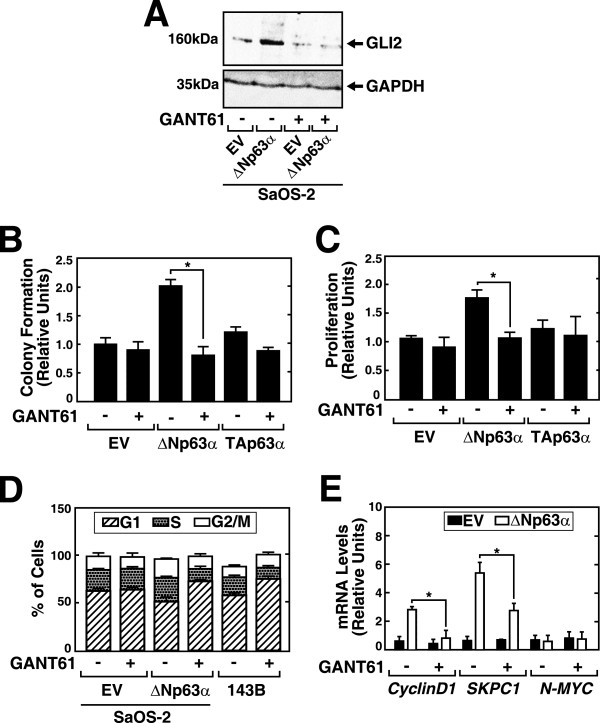
**GLI2 mediates the oncogenic potential of ΔNp63α in OS cells. A**. Cell lysates of SaOS-2-EV and SaOS-2-ΔNp62α cells untreated or treated with 20 μM GANT61 were analysed using western blotting with an anti- GLI2 antibody. **B**. GANT61 (20 μM) reversed enhanced anchorage-independent growth in SaOS-2-ΔNp62α cells determined using a soft agar assay and **(C)** proliferation was assessed using a WST assay. Data represent the mean ± SE of three independent experiments. *P value < 0.05. **D**. SaOS-2-EV, SaOS-2-ΔNp62α and 143B cells were treated with 20 μM GANT61 and subjected to cell cycle analysis 24 h after drug treatment. SaOS-2-ΔNp62α and 143B cells exposed to GANT61 were arrested in G1 phase. **E**. Real time PCR analysis of mRNA expression of cell-cycle related genes in SaOS-2-EV and SaOS-2-ΔNp62α cells. 24 h treatment with GANT61 reduced the levels of CyclinD1 and SKPC1 but not n-MYC. *P value < 0.05 (n = 3).

We next asked whether GLI2 mediated the oncogenic activities of ΔNp63α by treating SaOS-2-ΔNp63α cells with the small molecule GANT61 that specifically inhibit the binding of *GLI1 and GLI2* to DNA and inhibits downstream signaling through the Hh pathway [23]. GLI2 expression was significantly inhibited in SaOS-2-ΔNp63α cells treated with 20 μM GANT61 (Figure 6A). In contrast, the levels of ΔNp63α were not affected (Additional file 8).

We next tested whether inhibiting GLI2 expression with 20 μM GANT61 affected the enhanced colony formation and proliferation of SaOS-2-ΔNp63α cells. GANT61 treatment decreased colony formation by SaOS-2-ΔNp63α cells to the level of SaOS-2-EV and SaOS-2-TAp63 cells (Figure 6B). Moreover, GANT61 did not significantly inhibit colony formation by the latter two cell lines. Similarly, treatment of SaOS-2-ΔNp63α cells with GANT61 reduced their proliferation rate to that of SaOS-2-EV and SaOS-2-TAp63α cells (Figure 6C).

Treatment with GANT61 increased the percentage of cells in the G1 phase from 60.2 to 67.8%, from 52.6 to 75.6% and from 60.3 to 80.1% in SaOS-2-EV, SaOS-2-ΔNp63α and 143B cells respectively (Figure 6D). Although cell cycle arrest in SaOS-2-EV cells treated with GANT61 was not very prominent, the increase in the percentage of cells arrested in G1 phase was significantly higher in SaOS-2-ΔNp63α and 143B cells (Additional file 9).

Consistent with the cell cycle data, we found that treating SaOS-2-ΔNp63α cells with GANT61 decreased the expression of mRNAs encoding Cyclin D1 and SKP2 but not that of n-MYC. Treating SaOS-2-EV cells with GANT61 did not significantly change the levels of any of these transcripts (Figure 6E), suggesting that the expression of ΔNp63α and GLI2 regulates the cell cycle of OS with a more aggressive phenotype. Taken together, our findings demonstrate that the induction of *GLI2* expression by ΔNp63α mediates the oncogenic phenotype of OS cells.

## Discussion

Here, we studied the role of ΔNp63α in determining the oncogenic potential of OS cells and identified novel genes targeted by ΔNp63α. Initial studies of ΔNp63 led to the hypothesis that upregulation of its expression counteracts *p53* functions. However, evidence indicated that ΔNp63α induced the proliferation of cancer cells through the transactivation of certain target genes [24]. Yang *et al.* identified *p63* target genes that were involved in cell adhesion, proliferation, death, and were components of the Wnt and TGFβ signaling pathways [25]. *p63* has been implicated in cell adhesion programs involved in tumor invasion and cell migration [26]. Of all ΔNp63 isoforms, ΔNp63α appears to have the most potent effects, which may be attributed to the presence of sterile α motifs (SAM) and post SAM domains that recruit other proteins or fold up and inactivate the TA domains [7, 27].

ΔNp63α is overexpressed in squamous carcinomas, cancers of epithelial origin such as head and neck and lungs and may enhance cell growth by counteracting p53-mediated growth arrest and apoptosis [28]. Bid *et al.* suggest that ΔNp63α contributes to the progression of OS and neuroblastoma by inducing tumor angiogenesis, because they found that lung sections from patients with OS express high levels of ΔNp63α compared with the primary lesions from the same patients and that this promotes secretion of IL-6 and IL-8 to induce angiogenesis. They hypothesized that ΔNp63α provides the cells with a gain of function that leads to malignant transformation. Moreover, silencing of ΔNp63α expression in highly invasive OS cell lines significantly reduces proliferation and anchorage independent growth [10]. In their study elevated levels of ∆Np63 were detected in 57% of the OS samples; however, these data were not correlated to any clinical parameters. Our TMA analysis of expression of ∆Np63 in high-grade OS patients showed that prognosis was worse in resections while no significant correlation was found for ∆Np63 expression in biopsies.

Here, we report that ΔNp63α is the major p63 isoform expressed in OS cell lines and that *GLI2* is regulated by ΔNp63α to promote its oncogenic properties. By analysing human OS cell lines that differ in invasive properties, we found that ΔNp63α is overexpressed only in invasive OS cell lines. We further demonstrate that enforced expression of ΔNp63α in low metastatic SaOS-2 OS cell lines, which expressed low levels of endogenous p63 protein and mRNA, increased the cell proliferation rate, cell motility and enhanced anchorage independent growth. Because proliferation and colony formation are important cellular properties that contribute to the cascade that leads to metastasis, our findings underscore the importance of ΔNp63α expression in the malignant progression of OS.

SaOS-2-ΔNp63α cells did not undergo apoptosis (data not shown), in contrast to a report that ectopic expression of ΔNp63α in p53/p63 null non-small lung cancer cells induces apoptosis and cell cycle arrest [29]. However, our observation agrees with the results of a study of pancreatic ductal adenocarcinoma (PDAC) where overexpression of ΔNp63α in the p63 null PDAC cell line PANC-1 did not induce apoptosis [30]. King *et al.* reported that overexpression of ΔNp63α in mouse keratinocytes maintained proliferation under conditions that normally induce growth arrest and differentiation [31].

To enhance our understanding of pathogenesis of OS and to define the molecular pathways that are activated by ΔNp63α, we conducted microarray analysis to determine the transcription profile in SaOS-2 cells transfected with ΔNp63α expression vector. The search for p63 target genes identified many pathways regulated by p63 [32, 33], and most of the upregulated genes were identified in the present study. Analysis using DAVID grouped the up-regulated genes into clusters according to their Gene Ontology (GO) functions and identified components of Hh signaling pathway and GLI2 in particular as a novel transcriptional target of ΔNp63α.

Mohseny *et al.* reported that Hh pathway activity varied widely among OS cell lines and did not correlate with the patient survival [34]. However, Lo *et al.* analysed Hh pathway genes in 43 human primary high-grade OS samples and found that expression levels of genes encoding IHH, PTCH1 and GLI genes but not SMO were higher in tumors. Activation of ligand dependent (IHH-PTCH1 co-expression), and ligand independent (SMO, PTCH1, GLI) signaling might lead to Hh activation in OS. Moreover, the high levels of IHH may lead to larger tumor size, a prognostic factor of OS and indicate that activation of Hh signaling is required for OS progression [35]. *GLI2* expression is required for the growth of OS cells and *GLI2* is overexpressed in OS biopsy specimens and OS cell lines [36]. Inhibiting expression of *GLI2* in 143B cells transfected with *GLI2*- shRNA inhibited OS growth and reduced tumor volume 12 weeks after inoculation in nude mice [37].

The current study determined whether *GLI2* was regulated by ΔNp63α in OS. To investigate the functional association of ΔNp63α and *GLI2* in OS, we used siRNA technology to silence the expression of p63 in invasive OS cells that expressed high levels of endogenous ΔNp63α. Bid *et al.* demonstrated that silencing endogenous ΔNp63α in highly proliferating tumorigenic OS cell lines reduced cell proliferation and colony formation. Injecting nude mice with an invasive OS cell line transduced with a ΔNp63 expression vector reduced tumor formation compared with the control thereby establishing the importance of ΔNp63α in OS metastasis [10]. In the present study, inhibition of expression of ΔNp63α in invasive OS cells downregulated *GLI2* expression. Moreover, we determined that ΔNp63α binds directly to the *GLI2* promoter which explains how *GLI2* expression is regulated by ΔNp63α.

Using an inhibitor (GANT61) of *GLI2*, we showed here that GLI2 mediated the oncogenic effects of ΔNp63α. Because GANT61 inhibits *GLI1* as well as *GLI2* induced transcription, additional studies are required to determine the contributions of individual GLI family members to the malignant phenotype of OS. *GLI1* is overexpressed and *GLI2* is downregulated in SaOS-2 cells [38, 39]. Interestingly, SaOS-2 cells are also reported to express high levels of *GLI2* [40]. Although *GLI1* and *GLI2* function as transcriptional activators of the Hh pathway, they have nonredundant roles in osteosarcoma [38].

Taken together, our studies indicate an important role of the ΔNp63α-*GLI2* axis in OS progression. Our data warrant further investigations of treatment of patients with OS with high levels of *GLI2* expression with inhibitors of ΔNp63α signaling.

## Conclusions

In summary, ΔNp63α regulates the expression of *GLI2* and is involved in the metastasis of osteosarcoma. Our data contribute to a better understanding of the role of the transcription factors, ΔNp63α and *GLI2* in OS and suggest that the ΔNp63α-*GLI2* axis may serve as a target for therapy of osteosarcoma.

## Electronic supplementary material

Additional file 1: **List of primers used for the qRT- PCR experiments.** (PDF 57 KB)

Additional file 2: **Analysis of the specificity of the anti-ΔNp63 antibody.** Western blot analysis of whole cell extracts showing the reactivity of the anti- ΔNp63 antibody with cells transfected with TAp63α, TAp63γ, ΔNp63α, ΔNp63γ. (PDF 73 KB)

Additional file 3: **Analysis of ectopic and endogenous ΔNp63α and TAp63α proteins.** **A**. Western blot analysis of ΔNp63α in SaOS-2-EV, SaOS-2- ΔNp63α and 143B cells. **B**. Western blot analysis of TAp63α in SaOS-2-EV, SaOS-2-TAp63 and HOS cells. (PDF 214 KB)

Additional file 4: **Tissue Microarray of ΔNp63 in high grade OS samples A. Tissue cores representing the entire grading scheme used to score ΔNp63 staining.** B. Kaplan–Meier analysis. Patients with high-grade OS patients were divided into two groups based on the level of ΔNp63 . The prognosis of patients with <50% ΔNp63-positive tumor cells in tumor resections (grey line) was significantly higher compared with patients with high levels of ΔNp63 in resected tumor tissue (black line). (PDF 168 KB)

Additional file 5: **Western blot analysis of TAp63α and** ***GLI2*** **expression in lysates prepared from HOS and HU09 cells after transfection with control or** ***p63*** **siRNAs.** (PDF 60 KB)

Additional file 6: **Real time PCR analysis of** ***IFITM5, SMPD3*** **and** ***ECEL1*** **in 143B and M132 cells after transfection with control or** ***p63*** **siRNAs.** (PDF 29 KB)

Additional File 7: **The human** ***GLI2*** **locus illustrating the region detected using ChIP.** Sequence information of the promoter region is shown in detail, regions highlighted yellow correspond to sequences amplified using the GLI2-1 and GLI2-2 primer sets. The p63 consensus sequence present in the GLI2-1 amplified region is enclosed by the red rectangle. The bold and underlined bases are essential for GLI2 binding. The sequence of exon1 is enclosed by the polygon. (PDF 341 KB)

Additional File 8: **Protein levels of ΔNp63α in SaOS-2-ΔNp63α cells treated or untreated with 20 μM GANT61.** (PDF 132 KB)

Additional File 9: **Cell cycle analysis of 143B, SaOS-2-ΔNp63α and SaOS-2-EV cells treated or not treated with 20 μM GANT61.** Cell cycle analysis using FACS shows the percentage of cells in the G1, S and G2/M phases of 143B, SaOS-2-ΔNp63α and SaOS-2-EV cells treated with 20 μM GANT61. (PDF 1 MB)
