# Supplementary material for: ΔNp63α enhances the oncogenic phenotype of osteosarcoma cells by inducing the expression of GLI2
Source: BMC Cancer. 2014 Aug 1;14:559. doi: 10.1186/1471-2407-14-559 (PMC4125704; doi:10.1186/1471-2407-14-559)
Supplement: Supplementary file 1 — Additional file 1: List of primers used for the qRT- PCR experiments. (PDF 57 KB) [file 12885_2014_4737_MOESM1_ESM.pdf]

<u>Additional File 1: Primers used for real- time PCR analysis</u>

GLI-2

Forward primer: 5-CGACACCAGGAAGGAAGGTA-3

Reverse primer: 5-AGAACGGAGGTAGTGCTCCA-3

IFITM5

Forward primer: 5´CCC GCC TCG AGA CCA CTT GA´3

Reverse primer: 5´GCC GCT TCC AGG TCA CCA AC´3

PRSS56

Forward primer: 5´ CCCGAATGAGCTTCTGTGGA´3

Reverse primer: 5´ CAGGAGGTGACTCCGAACAG´3

ECEL1

Forward primer: 5´ CTCTCAACTACGGGGGCATC´3

Reverse primer: 5´ GCATGCTTGTCAGTCAGCAC ´3

SMPD

Forward primer: 5’ CGA CTC ACT CGC CAG GGT CA´3

Reverse primer: 5’ AGC GGC GCT GAT GGA GGT AT´3

CDH15

Forward primer: 5’CTG AAC GTG ACC GTG TGC CG´3

Reverse primer: 5’GGC CAG CAC GAT GAC CAG TG´3

MFAP5

Forward primer: 5’ GGGTCAATAGTCAACGAGGAGAC´3

Reverse primer: 5’ GCCAAGTCATCTGTGGAAGGTG´3

CXCL12

Forward primer: 5’ CTCAACACTCCAAACTGTGCCC´3

Reverse primer: 5’CTCCAGGTACTCCTGAATCCAC´3

RAMP1

Forward primer: 5’ CTCACCCAGTTCCAGGTAGACA´3

Reverse primer: 5’ CAGGAAGAACCTGTCCACCTCT´3

CPA4

Forward primer: 5’ CAATGAAGGGCAAGAACGGAGC´3

Reverse primer: 5’ GGTCAGGAAAGTCTGCGGCAAT´3

NTNG1

Forward primer: 5’ GCACGCTACTTTTACGCGATCTC´3

Reverse primer: 5’ CTGGACCTGTAGTGTTGTGCTC´3

CyclinD1

Forward primer: 5’TGC CTG TAC TGA GCT GGG CAA´3

Reverse primer: 5’TGG CTG CAG AAG AGG GTG TTG´3

SKP2

Forward primer: 5’ GCT GCT AAA GGT CTC TGG TGT´3

Reverse primer: 5’ AGG CTT AGA TTC TGC AAC TTG´3

n-MYC

Forward primer: 5’ CTT CGG TCC AGC TTT CTC AC´3

Reverse primer: 5’ GTC CGA GCG TGT TCA ATT TT´3

GAPDH

Forward primer: 5’ TGAACGGGAAGCTCACTGGCATGG´3

Reverse primer: 5’ TGGGTGTCGCTGTTGAAGTCAGAGGAGA´3
