# Supplementary figures and images for: ΔNp63α enhances the oncogenic phenotype of osteosarcoma cells by inducing the expression of GLI2
Source: BMC Cancer. 2014 Aug 1;14:559. doi: 10.1186/1471-2407-14-559 (PMC4125704; doi:10.1186/1471-2407-14-559)

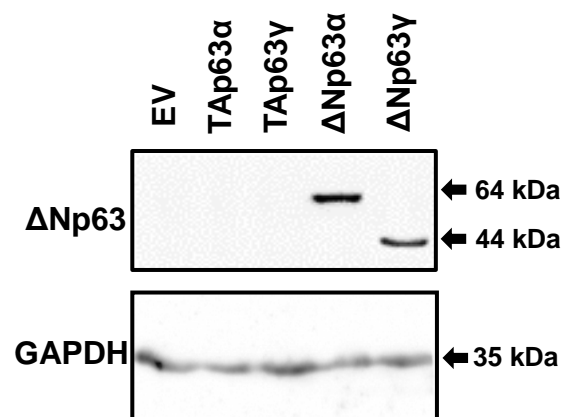

Supplement: Supplementary file 2 — Additional file 2: Analysis of the specificity of the anti-ΔNp63 antibody. Western blot analysis of whole cell extracts showing the reactivity of the anti- ΔNp63 antibody with cells transfected with TAp63α, TAp63γ, ΔNp63α, ΔNp63γ. (PDF 73 KB) [file 12885_2014_4737_MOESM2_ESM.pdf]

A

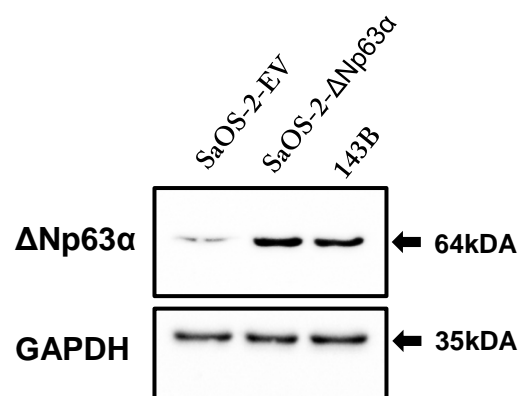

B

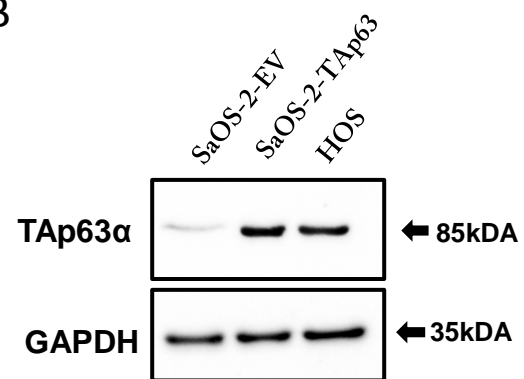

Supplement: Supplementary file 3 — Additional file 3: Analysis of ectopic and endogenous ΔNp63α and TAp63α proteins. A. Western blot analysis of ΔNp63α in SaOS-2-EV, SaOS-2- ΔNp63α and 143B cells. B. Western blot analysis of TAp63α in SaOS-2-EV, SaOS-2-TAp63 and HOS cells. (PDF 214 KB) [file 12885_2014_4737_MOESM3_ESM.pdf]

A

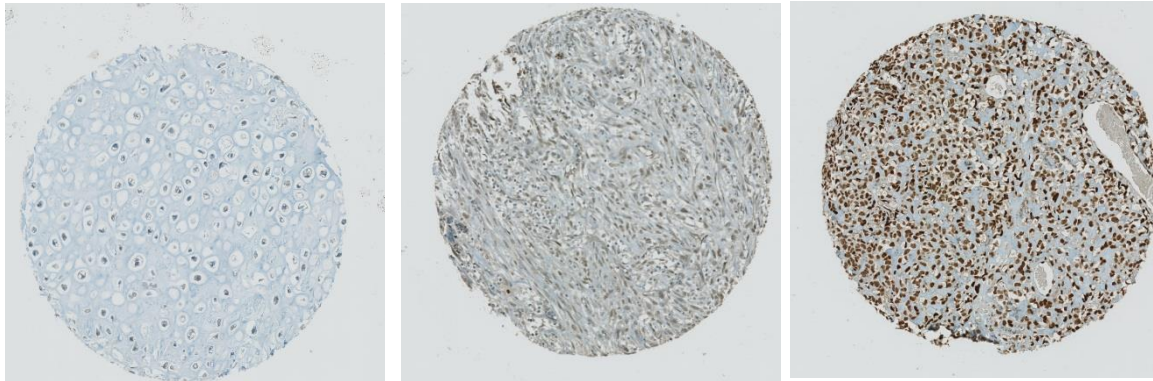 $\Delta$ Np63 negative $\Delta$ Np63 low expression $\Delta$ Np63 high expression

B

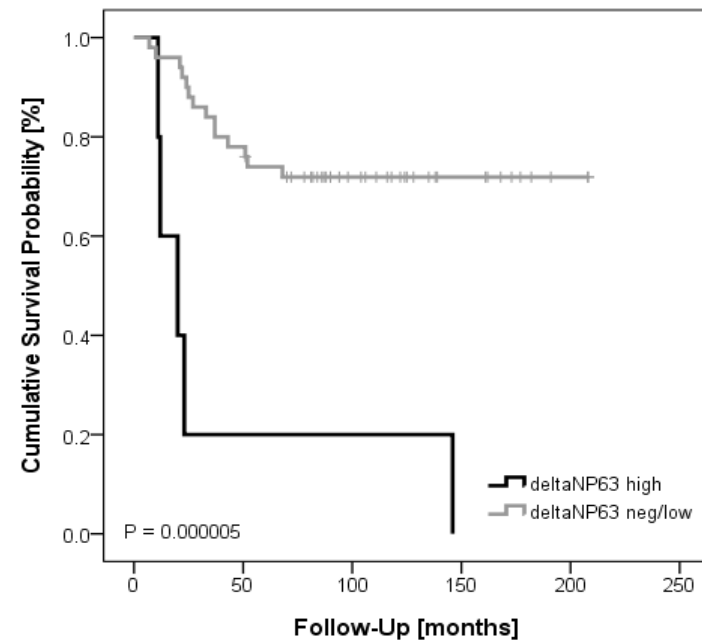

Supplement: Supplementary file 4 — Additional file 4: Tissue Microarray of ΔNp63 in high grade OS samples A. Tissue cores representing the entire grading scheme used to score ΔNp63 staining. B. Kaplan–Meier analysis. Patients with high-grade OS patients were divided into two groups based on the level of ΔNp63 . The prognosis of patients with <50% ΔNp63-positive tumor cells in tumor resections (grey line) was significantly higher compared with patients with high levels of ΔNp63 in resected tumor tissue (black line). (PDF 168 KB) [file 12885_2014_4737_MOESM4_ESM.pdf]

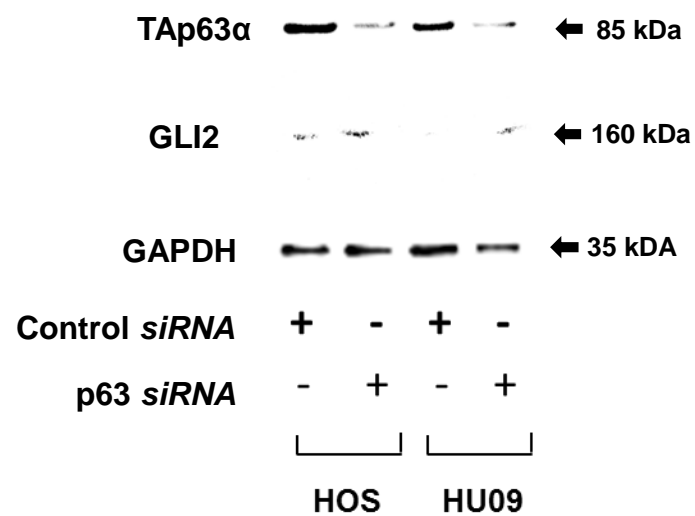

Supplement: Supplementary file 5 — Additional file 5: Western blot analysis of TAp63α and GLI2 expression in lysates prepared from HOS and HU09 cells after transfection with control or p63 siRNAs. (PDF 60 KB) [file 12885_2014_4737_MOESM5_ESM.pdf]

## Additional file 6

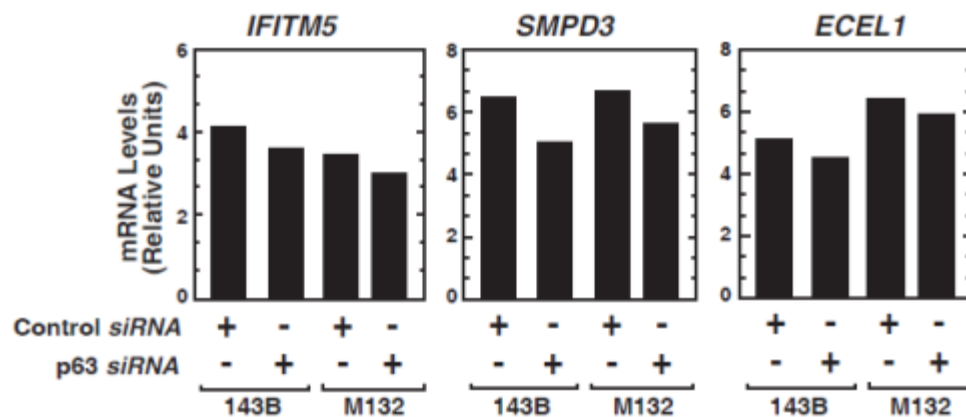

Supplement: Supplementary file 6 — Additional file 6: Real time PCR analysis of IFITM5, SMPD3 and ECEL1 in 143B and M132 cells after transfection with control or p63 siRNAs. (PDF 29 KB) [file 12885_2014_4737_MOESM6_ESM.pdf]

Additional File 7

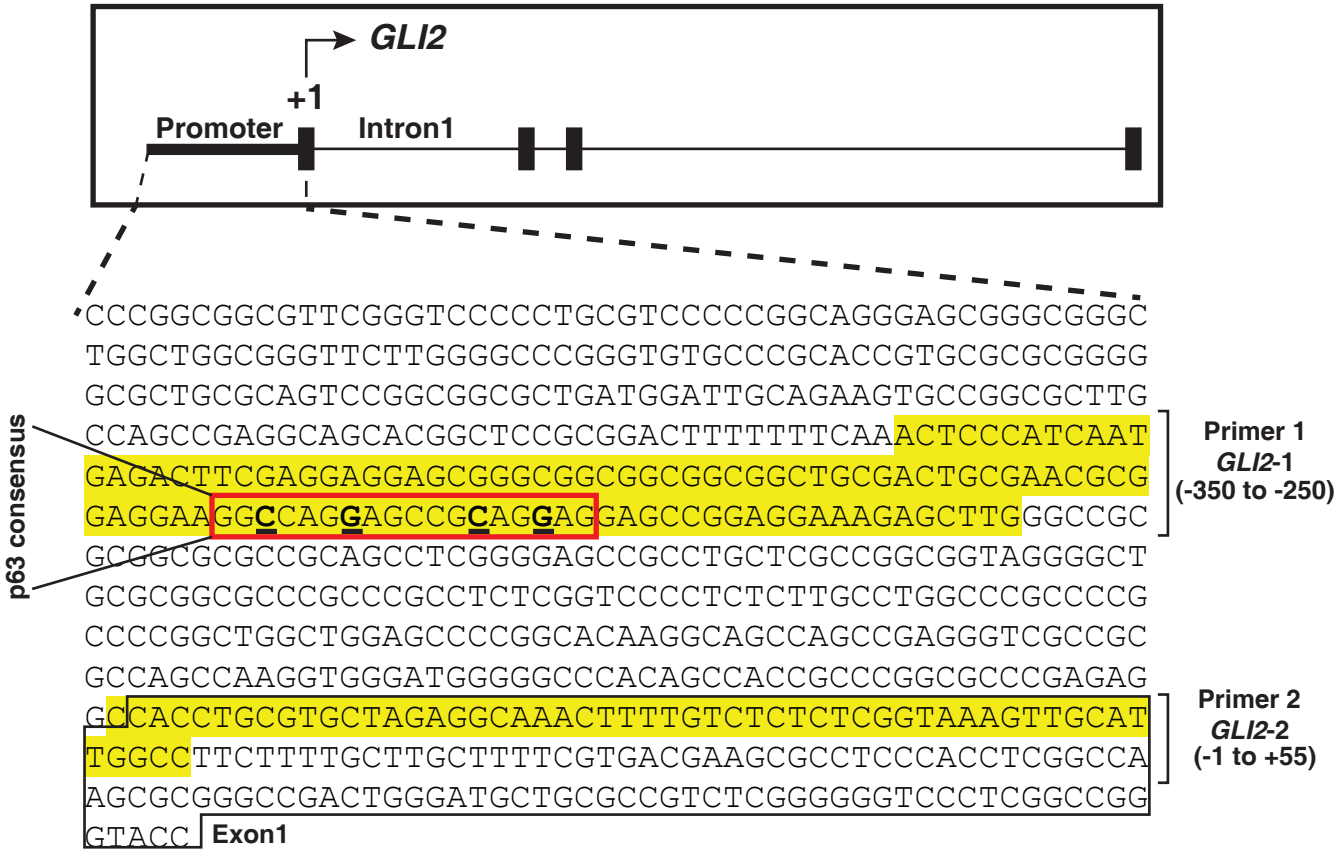

Supplement: Supplementary file 7 — Additional File 7: The human GLI2 locus illustrating the region detected using ChIP. Sequence information of the promoter region is shown in detail, regions highlighted yellow correspond to sequences amplified using the GLI2-1 and GLI2-2 primer sets. The p63 consensus sequence present in the GLI2-1 amplified region is enclosed by the red rectangle. The bold and underlined bases are essential for GLI2 binding. The sequence of exon1 is enclosed by the polygon. (PDF 341 KB) [file 12885_2014_4737_MOESM7_ESM.pdf]

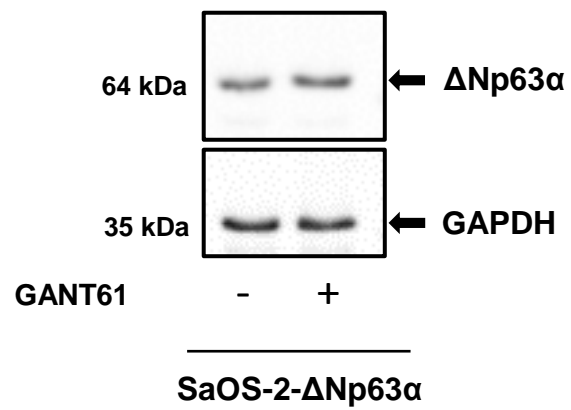

Supplement: Supplementary file 8 — Additional File 8: Protein levels of ΔNp63α in SaOS-2-ΔNp63α cells treated or untreated with 20 μM GANT61. (PDF 132 KB) [file 12885_2014_4737_MOESM8_ESM.pdf]

# Additional File 9

143B

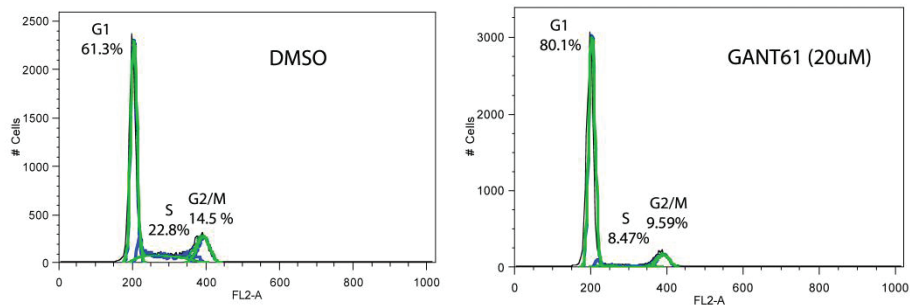

SaOS-2-EV

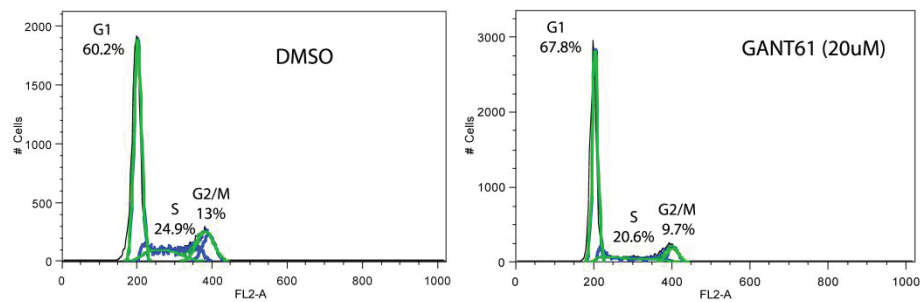

SaOS-2-ΔNp63α

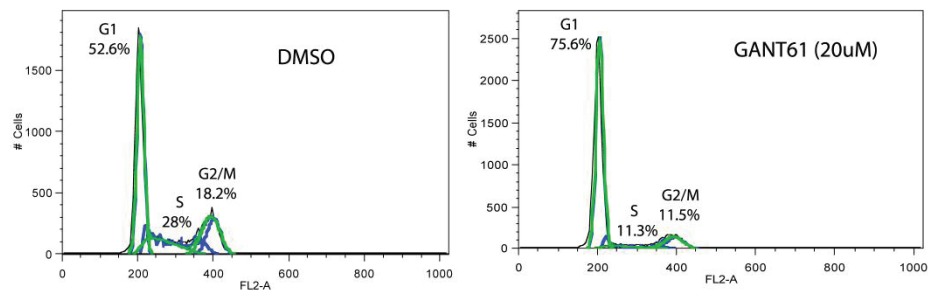

Supplement: Supplementary file 9 — Additional File 9: Cell cycle analysis of 143B, SaOS-2-ΔNp63α and SaOS-2-EV cells treated or not treated with 20 μM GANT61. Cell cycle analysis using FACS shows the percentage of cells in the G1, S and G2/M phases of 143B, SaOS-2-ΔNp63α and SaOS-2-EV cells treated with 20 μM GANT61. (PDF 1 MB) [file 12885_2014_4737_MOESM9_ESM.pdf]
